# Individual to Community-Level Faunal Responses to Environmental Change from a Marine Fossil Record of Early Miocene Global Warming

**DOI:** 10.1371/journal.pone.0036290

**Published:** 2012-04-27

**Authors:** Christina L. Belanger

**Affiliations:** Department of the Geophysical Sciences, University of Chicago, Chicago, Illinois, United States of America; Texas A&M University, United States of America

## Abstract

Modern climate change has a strong potential to shift earth systems and biological communities into novel states that have no present-day analog, leaving ecologists with no observational basis to predict the likely biotic effects. Fossil records contain long time-series of past environmental changes outside the range of modern observation, which are vital for predicting future ecological responses, and are capable of (a) providing detailed information on rates of ecological change, (b) illuminating the environmental drivers of those changes, and (c) recording the effects of environmental change on individual physiological rates. Outcrops of Early Miocene Newport Member of the Astoria Formation (Oregon) provide one such time series. This record of benthic foraminiferal and molluscan community change from continental shelf depths spans a past interval environmental change (∼20.3-16.7 mya) during which the region warmed 2.1–4.5°C, surface productivity and benthic organic carbon flux increased, and benthic oxygenation decreased, perhaps driven by intensified upwelling as on the modern Oregon coast. The Newport Member record shows that (a) ecological responses to natural environmental change can be abrupt, (b) productivity can be the primary driver of faunal change during global warming, (c) molluscs had a threshold response to productivity change while foraminifera changed gradually, and (d) changes in bivalve body size and growth rates parallel changes in taxonomic composition at the community level, indicating that, either directly or indirectly through some other biological parameter, the physiological tolerances of species do influence community change. Ecological studies in modern and fossil records that consider multiple ecological levels, environmental parameters, and taxonomic groups can provide critical information for predicting future ecological change and evaluating species vulnerability.

## Introduction

The fossil record is a key source of data on biotic responses to past and ongoing climate change and its importance to establishing natural baselines of environmental and ecological change are increasingly realized [1]–[3]. Fossil records also permit long-term studies of ecological responses to environmental changes over millennia to millions of years – time scales unobtainable by modern ecological studies. Fossil records that pre-date human influences are especially valuable because the effects of climate change can be assessed without confounding anthropogenic factors such as pollution, eutrophication, fishing, and human-facilitated invasive species. In addition, future environmental conditions and ecosystem states may have no present-day analog [4]–[5]. Investigating past conditions can broaden the range of observed ecosystem states and increase our ability to predict the behavior of systems outside modern observation.

Coastal marine environments need critical study because they are highly productive systems rich in biodiversity and important human resources. Coastal environments are also especially vulnerable to climate change and have experienced greater environmental changes with modern global warming than open ocean settings [6]–[7]. Marine benthic communities from subtidal soft-sediment environments contain taphonomically durable taxa, like benthic foraminifera and mollusks, which provide excellent fossil time-series of community structure and composition with high fidelity to the original, living, assemblages [8]–[10]. These marine benthic groups are thus valuable for examining past ecological change in coastal systems.

In addition to illuminating ecosystem states with no present-day analog, fossil records can reveal the tempo of past faunal responses, which are important to predicting modern ecological changes. Temporal changes in ecological communities may occur gradually or have stepped, pulsed shifts that are rapid compared to ecological dynamics before and after the shift [11]. Large, sudden shifts across an ecological threshold may be difficult to reverse if the new community represents an alternative stable state or ecological regime [11]–[13]. Much of environmental management, however, is built upon a model of gradual ecosystem changes [14]. Furthermore, documented shifts to alternative stable states in the marine realm often result from, or at least occur in the context of, direct human influence (ie. eutrophication or over-fishing) rather than anthropogenic climate change alone [15]. Fossil records that pre-date human impacts are thus essential testing grounds for determining if abrupt, threshold, shifts are a general mode of biological response to climate change.

Many studies of biotic responses to climate change focus on temperature, but other stressors such as changes in productivity and benthic oxygenation that coincide with temperature change can play a major role in marine benthic community structure [16]–[19]. Changes in productivity and oxygenation can be linked to temperature change, but these environmental properties can be variably coupled or decoupled so that temperature itself may not be the direct driver of biotic responses [20]–[22]. Proxies, such as δ^18^O (temperature and salinity) and δ^13^C (productivity) derived from benthic foraminiferal tests and information on bottom water oxygenation and water energy from sedimentology, can provide climate data for fossil records and allow us to identify the proximal drivers of marine benthic faunal change.

Further complicating the analysis of biotic responses, both in modern and ancient settings, is that different taxonomic groups may respond to the same environmental change but in different ways, may respond most strongly to different environmental parameters, or may respond at different thresholds of change. For example, molluscs and benthic foraminifera commonly co-occur and have fossil records preserved in the same sediments, but interact with different aspects of the environment. Examining multiple taxonomic groups from the same fossil record provides a test of the generality of faunal responses, and the environmental factors that drive those faunal changes, despite biological disparity.

Paleoecological studies rarely examine multiple ecological levels, but studying the community, population, and individual levels in a single fauna can lead to an understanding of the biological mechanisms underlying faunal changes. For example, physiological limits are often used to explain the geographic range shifts of marine organisms that are associated with climate change [23]–[25], but this is rarely tested in paleoecological studies and few studies consider both processes at multiple levels. However, in organisms that grow by accretion, we can use growth banding and stable isotope sclerochronology in fossil material to reconstruct growth rates, which are a proxy for physiological rates, and thus directly test whether physiological limits underlie faunal change. If body size and growth rates decline in species whose abundances decline during an environmental change, we can infer that community-level change is driven, at least in part, by mechanisms at the level of individual physiological tolerances. Alternative hypotheses for climate-linked biotic change within the marine realm include changes in settlement or recruitment potentials [16], [26] and changes in ecological interactions [27]–[28].

To extract biotic responses and their environmental drivers at the individual, population, and community levels from past intervals of long-term climate change, we need fossil records that (a) were deposited across past climate change events, (b) can be analyzed at high enough resolution to evaluate trends in regional faunal and environmental changes, and (c) have fossils well enough preserved for geochemical and growth line analyses. Here I present an example of the utility of fossil records that fit these criteria using a rich record of co-occurring benthic foraminifera and molluscs from the Early Miocene Newport Member Astoria Formation exposed in coastal cliffs along central Oregon (see Table 1 for faunal variables measured). This record shows that (a) ecological responses to natural environmental change can be abrupt, (b) productivity can be the primary driver of faunal change during global warming, (c) molluscs and foraminifera respond differently to the same environmental changes, and (d) physiological mechanisms underlie community-level change.

**Table 1:** Faunal variables measured in the Newport Member of the Astoria Formation; source indicates the materials measured for each variable.

Faunal Variable	Ecological Level	Source	Ecological Metric
Species Richness	Community	Molluscan and Foraminiferal bulk samples	sample size standardized number of species
Evenness	Community	Molluscan and Foraminiferal bulk samples	probability of intraspecific encounter (PIE)
Relative abundance of ecologically distinct groups	Community	Molluscan, Foraminiferal bulk samples	proportional abundance of subsurface deposit-feeding bivalves and organic-loving benthic foraminifera
Taxonomic Composition	Community	Molluscan and Foraminiferal bulk samples	Ordination score (DCA, NMDS)
Average Body Size 1	Community	Bivalves	geometric mean size of valve for all bivalve taxa
Average Body Size 2	Population	Bivalves	geometric mean size of valve for bivalve species
Shell growth rate	Individual	Bivalves	distance between external growth bands, internal growth bands, or δ^18^O maxima
Age at first reproduction*	Individual	Bivalves	reproductive growth checks; mismatch between “annual” growth bands and δ^18^O

* = Variables not measured in the present study, which have the potential to yield additional information after methodological improvements.

### Geological and Environmental Background

The Early Miocene was an interval of increasing temperature leading into the Middle Miocene Climate Optimum, the warmest time in the past 20 million years [29]. Earlier work on the paleoenvironmental changes in the Newport Member using the same samples described herein documents regional warming on the order of 2.1 to 4.5°C between 19.5 and 18.5 Ma; the total ∼80 m thick stratigraphic record spans ∼20.3-16.7 mya [30]. In addition to temperature change, surface water productivity and organic carbon flux to the seafloor both increased at ∼19 mya, resulting in dysoxic benthic conditions [30].

The stratigraphic section was deposited in a structural embayment on the Miocene Oregon coast [31] and is dominated by middle to outer shelf siliciclastic sandy-mud facies. Most of the section is composed of mixed-grain populations of fine sand and mud, but moderately well-sorted fine to coarse sands and laminated muds are common in the upper 50 m of section (after ∼18.3 Ma). In the lower 30 m of the section (before ∼18.9 Ma), the medium gray strata are pervasively burrow-mottled with meter-scale bedding, but the upper 50 m is comprised of centimeter- to decimeter-scale bedding with dark gray silt and clay laminations in many beds. Sedimentary accumulation rates are ∼16.5 m/myr from 20.3 to 19 Ma then increase to ∼29 m/myr between 18.8 and 18 Ma [30].

Estimated absolute water depths are ∼25 m based on the molluscan fauna [32]–[34] and <150 m based on the benthic foraminifera fauna [34]. Hummocky cross-stratification preserved in tuff beds and in mud-capped sandy beds are found at stratigraphic levels throughout the section, further supporting deposition in shelf water depths, specifically between the lower shoreface and storm wave base (e.g., [35]). This restricted depth of deposition throughout the section ensures that faunal assemblages in the time series are drawn from similar environments.

**Figure 1:**
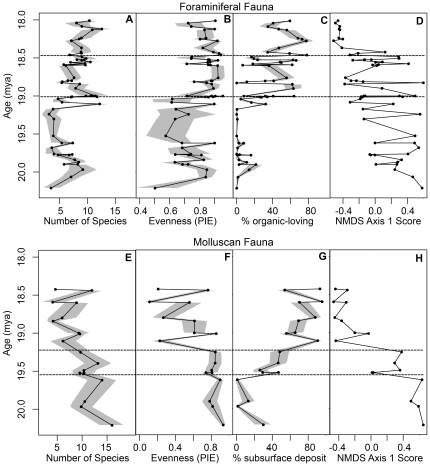
Changes in community structure over time. (A) Foraminiferal species richness, (B) foraminiferal community evenness, (C) the proportion of organic-loving foraminifers, (D) NMDS axis 1 ordination scores from foraminiferal species abundances, (E) molluscan species richness, (F) molluscan community evenness, (G) the proportion of subsurface deposit-feeding bivalves, and (H) NMDS axis 1 ordination scores from molluscan species abundances. Gray shading indicates the 95% confidence intervals of the ecological metrics following sample size standardization. Dashed lines indicate the boundaries between communities (Table 2).

Fossil collections are from a ∼5 km stretch of coastline from Beverly Beach State Park to Newport, Or (see [30] for location map and stratigraphic log). Fossil densities are highest in the lower 54 m of the section (∼20.3-18 mya) and only one molluscan and one foraminiferal faunal count were recovered from sediments younger than 18 Ma (Table S1). This younger sample (∼16.7 Ma) is included in analyses, but is excluded from Figure 1 for clarity. Fossiliferous beds are typically poorly sorted very-fine to fine sandstones; specimens from coarser sediments (intervals 31–35 m and 55–62 m above the base of the section) are excluded from analysis because the sedimentology and faunal composition suggest a sandy shoreface environment distinct from the shelf depths inferred for the rest of the section.

The molluscan fauna is preserved as original aragonite and calcite shells and are taphonomically comparable throughout the section. Bivalves are preserved as whole to large fragments (umbo included) and are articulated and closed in ∼50% of specimens. Occasionally, valves are preserved articulated but open and many specimens are disarticulated but found in associated valve pairs. This suggests minimal transport and disruption of fossil material. Preservation quality in the total foraminiferal assemblage varies among samples, but differences in sample quality are largely due to changing abundances of thin-walled species and do not affect paleoecological interpretations [36].

## Results and Discussion

### Changes in community structure and composition are abrupt

For both benthic foraminiferal (n = 58) and molluscan (n = 19) time series, faunal composition changes in steps that are abrupt with respect to the duration of each distinct assemblage or community state (Figure 1). Among foraminiferal assemblages, species richness has two statistically supported steps (a decrease at 19.60 and an increase at 19.17 Ma; see Methods and Table S2), community evenness has one supported increase (at 19.03 Ma), and two increases in the proportional abundances of dysoxic taxa are supported (at 19.03 and 18.48 Ma). Among molluscan assemblages, species diversity and community evenness decrease once at 19.25 Ma, average body size decreases in two steps (at 19.50 and 19.25 Ma), and the proportional abundance of subsurface deposit feeders increases in two steps (at 19.60 and 19.25 Ma). Abrupt shifts in species richness, evenness, body size, and the proportion of ecologically distinctive taxa appear to be coordinated within each taxonomic group allowing each faunal time series to be divided into three distinct community states (Table 2). Each step is instantaneous at the resolution of sampling (<0.1 Ma for the foraminifera and <0.3 Ma for the molluscs); changes between communities may in fact be gradual on finer timescales.

**Table 2:** Community states recognized in benthic foraminiferal and molluscan assemblages based on shifts in species richness, evenness and taxonomic composition.

Foraminiferal Community	Age Range (mya)	Median Richness (IQR)	Median Evenness (IQR)	Description
C	18.48-16.71	9.00 (7.74–9.50)	0.77 (0.72–0.81)	Semi-infaunal *Bolivina astoriensis* and fully-infaunal *Buliminella bassendorfensis* and *Nonionella* spp. each >20% of individuals. Semi-infaunal *Buccella mansfieldi* and fully-infaunal *Bolivina ovata and Fursenkoina punctata* >5% each. Organic-loving taxa together comprise 60% of individuals.
B	19.03-18.48	8.98 (7.10–10.56)	0.77 (0.72–0.79)	*B. astoriensis and Pseudononion costiferum* (fully infaunal) each >20% of individuals. *B. mansfieldi and B. bassendorfensis* each >10%. Organic-loving taxa together comprise 33%.
A	20.26-19.03	5.72 (4.16–8.05)	0.56 (0.49–0.7)	Characterized by *B. astoriensis* (44% of individuals). *B. mansfiedli* and *P. costiferum* each >10% of individuals. Organic-loving taxa together comprise 7%.

Molluscan Community	Age Range (mya)	Median Richness (IQR)	Median Evenness (IQR)	Description
3	19.25-16.71	6.70 (5.12–10.30)	0.38 (0.19–0.55)	Subsurface deposit-feeder *Saccella* spp. >78% of individuals. Filter-feeder *Anadara devincta* and surface deposit-feeder *Macoma albaria* each >3%.
2	19.6-19.25	11.35 (11.10–11.51)	0.74 (0.74–0.77)	Surface deposit-feeders *M. albaria* and *Macoma arctata*, and subsurface deposit-feeders *Acila conradi* and *Saccella* spp. each >10% of individuals. Filter-feeders A. devincta and *Katherinella augustifrons* each >3%.
1	20.26-19.6	14.21 (12.00–16.45)	0.80 (0.74–0.86)	Predatory scaphopods >20% of individuals. Filter-feeder *Chione ensifera* and surface-deposit feeder *Macoma arctata* each >15%. Subsurface deposit feeders *A. conradi* and *Litorhadia astoriana* and predatory gastropods *Cryptonatica oregonensis* and *Sinum scopulosum* each >3%.

IQR = interquartile range.

Offsets in the timing of steps between the foraminiferal and molluscan data sets (Figure 1) likely result from slight differences in sample ages and differences in sampling resolution (beds rich in molluscs are not always rich in benthic foraminifera and vice versa). The general coordination of steps suggests that both faunas are responding to an environmental change occurring at the same time. However, not all faunal shifts are coordinated between the two faunas. For example, the shift between molluscan Communities 1 and 2 is not reflected in the foraminiferal community nor is the shift between foraminiferal Communities B and C reflected in the molluscan fauna. Such offsets suggests that the two faunas are responding to different environmental parameters for at least some faunal changes or are responding at different threshold values of the same factor.

### Abrupt changes are characterized both by threshold responses and linear tracking of productivity

Despite some similarity in the timing of community steps, the directionality of changes in ecological metrics differs between the molluscan and foraminiferal time series. In the molluscs, species richness and evenness decline, whereas in the foraminifera species richness and evenness increase (Figure 1). On the other hand, species that favor organic rich environments increase in proportional abundance in both groups (Figure 1) suggesting that productivity is a key driver of ecological change in both groups. Increased upwelling during Miocene warming has been observed in other coastal settings [37]–[39] and could also have driven increased productivity on the Miocene Oregon Coast [30]. In addition, intensification of wind-driven upwelling is thought to drive seasonally dysoxic conditions on the modern Oregon Coast [40]–[41]; dysoxia is recognized in the Newport Member via a stratigraphic decrease in bioturbation and by the presence of well-laminated sediments after the shift to a high-productivity environment, as indicated by δ^13^C [30].

**Figure 2:**
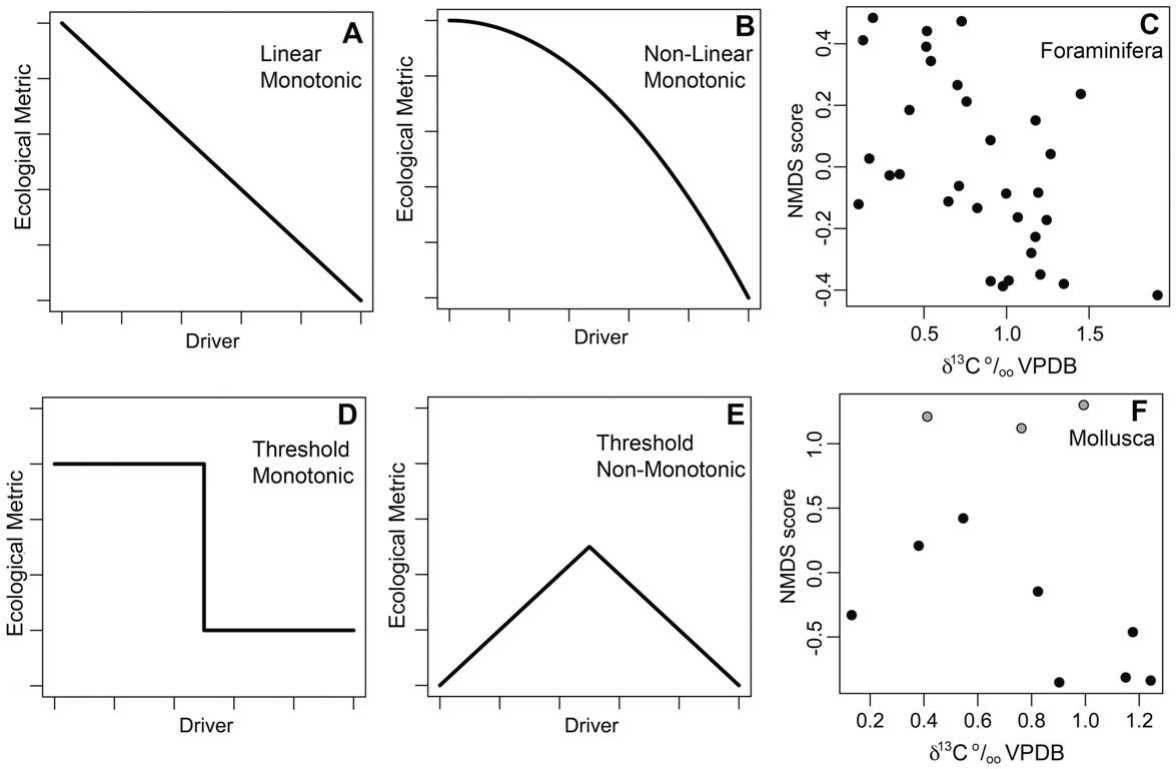
Relationship between ecological variables and environmental drivers. Models of relationships between ecological variables and environmental drivers (A–D). Observed relationship between NMDS ordination score and productivity (δ^13^C) in (E) foraminifera and (F) molluscs. Gray points in (F) depict Molluscan Community 1 and black depict Molluscan Communities 2 and 3. The foraminiferal relationship most closely resembles model A. The molluscan relationship most closely resembles model D.

The relationship between ecological metrics and environmental factors reveals whether step changes in ecological metrics are due to abrupt changes in underlying environmental changes or due to crossing ecological thresholds (Figure 2) [13]. Foraminiferal ecological metrics have approximately linear relationships with geochemical proxies for surface productivity and organic carbon flux (δ^13^C and Δδ^13^C respectively) suggesting that foraminiferal community structure is closely tracking productivity changes and that the abrupt ecological changes occur due to abrupt changes in the environment (Figure 2). This relationship to productivity is reflected in strong negative rank order correlations between measures of foraminiferal community composition and the environmental proxies δ^13^C and Δδ^13^C (Table 3). Species richness and community evenness do not have a strong relationship with any of the measured environmental variables.

**Table 3:** Partial correlations based on Spearman rank order between foraminiferal ecological and environmental variables.

	Richness	Evenness	NMDS 1	DCA 1	% Organic-loving
δ^18^O	−0.18	−0.255	0.174	0.317	−0.361*
δ^13^C	0.264	0.174	−0.325	−0.447**	0.537**
Δδ^13^C	−0.029	−0.006	−0.787***	−0.458**	0.383*
% mud	0.058	0.315	−0.132	−0.19	0.267

* p<0.05,

** p<0.01,

*** p<0.001.

Molluscan ecological metrics in Communities 2 and 3, on the other hand, have non-linear and non-monotonic relationships to surface productivity (Figure 2). Multivariate ordination axis 1 scores of taxonomic composition increase with increasing δ^13^C until a value of ∼0.8‰ at which point axis scores decrease sharply as δ^13^C value continue to rise. PIE also increases as δ^13^C increases from ∼0.1 to 0.8‰, but then decreases strongly as δ^13^C increases above ∼0.8‰. Low species diversity is only observed at the highest δ^13^C values. This pattern indicates a threshold level of productivity, or a related environmental factor such as benthic oxygenation, that triggers a major reorganization in the molluscan community. In contrast, the average community-level body size has an approximately linear relationship with δ^13^C, reflected in a strong negative correlation between body size and δ^13^C (body size decreases as productivity increases; Table 4). The relationship between productivity and body size evident in Molluscan Communities 2 and 3 is paralleled by Community 1, but differs due to differences in taxonomic composition (e.g. the dominate deposit-feeder is *Macoma arctata* and the dominate suspension-feeder is *Chione ensifera* in Community 1, but in Communities 2 and 3 the dominate deposit-feeder is *Saccella* spp. and the dominate suspension feeders are *Anadara devincta* and *Katherinella augustifrons*; Table 2).

**Table 4:** Partial correlations based on Spearman rank order between molluscan ecological and environmental variables.

	Richness	Evenness	NMDS 1	DCA 1	% Deposit-feeding	Body Size
δ^18^O	0.152	0.712*	0.419	0.616*	−0.406	0.287
δ^13^C	−0.063	−0.28	−0.148	−0.299	0.293	−0.816**
Δδ^13^C	−0.152	−0.372	−0.271	−0.423	0.302	−0.393
% mud	−0.37	−0.107	−0.452	−0.329	0.382	−0.482

* p<0.05,

** p<0.01,

*** p<0.001.

Molluscan community evenness and the detrended correspondence analysis axis 1 scores of taxonomic composition are significantly positively rank ordered with δ^18^O after accounting for mutual correlations with sample age (Table 4). This suggests either a temperature or a salinity driver for changes in the molluscan fauna. However, the molluscan and foraminiferal assemblages represent fully marine communities and the latitudinal ranges of modern congeners do not indicate an increase in equatorward (warm water) taxa between successive communities; the median modern latitude over which the majority of genera overlap is similar among communities (Figure S1). Thus neither a temperature or salinity driver is compelling. Instead, these correlations with δ^18^O may be a spurious consequence of sampling resolution: when the foraminiferal data set is culled to match the resolution of the molluscan data set, δ^18^O also appears more important despite the strong relationship to carbon cycle processes evident in the full foraminiferal data set (Table S3). The correlations between δ^18^O and molluscan ecological metrics are also less significant than environmental correlates with the foraminiferal metrics and could be type 1 errors.

Differences in the timing, tempo, and directionality of faunal changes between the molluscan and foraminiferal communities could be explained by differences in the temporal scale of community turnover. Foraminiferal lifespans are much shorter than most molluscs. For example, *Nonion depresulus* lives for only 3–4 months during which time it reproduces once [42]. Larger benthic foraminifera have longer life spans, but even for these taxa longevity is only between 4 months to two years [43]. These short life spans allow populations to respond rapidly to favorable conditions and complete their life cycles before those conditions vanish [44]. In addition, foraminiferal propagules can remain dormant in sediments for at least 2 years until favorable conditions return, increasing the rapidity with which foraminifera can respond to environmental change [45]–[46]. For example, *Nonion depresulus* is abundant in spring and fall coinciding with diatom blooms in coastal waters, but has very low abundances in the winter and summer when food resources are lower [42]. Standing crops also vary in parallel with upwelling in northern California in *Gabratella ornatissima* [47], and vary with phytoplankton blooms in many Southern California margin taxa [48]. Seasonal fluctuations in the standing crops of shallow benthic foraminifer species appear to be a general rule [44]. When time averaged in the rock record, these fluctuations between populations that are favored at different times of year, or in different years, would yield a more diverse and more even assemblage [49]–[50]. Thus, the increase in community evenness among the benthic foraminifera may arise from natural time-averaging of strong seasonality in environmental conditions and intra-annual community turnover rather than reflect the diversity that would be captured in a single sample of standing populations. Increased time-averaging up-section could also increase richness and evenness in the foraminiferal fauna, however, if variation in time-averaging was a major factor molluscan richness and evenness should co-vary with foraminiferal metrics.

In contrast to foraminifera, molluscs typically live multiple years with a median maximum lifespan of 8–9 years and may not reproduce in their first year of life [51]. Seasonality in the composition of molluscan communities is common among juvenile individuals due to the timing of reproductive events, but this is not reflected in shell assemblages drawn only from adult specimens >2 mm [52]. Only shells >4 mm are used in the present study.

With eutrophication, marine benthic species dominance tends to shift from K to r reproductive strategies [53]–[54] suggesting that foraminifera should generally fare better than molluscs during a rise in productivity. Low-oxygen tied to high productivity could also ultimately explain the observed differences between the foraminiferal and molluscan communities in their relationship to productivity. Metazoans that live in well-oxygenated conditions exhibit oxygen stress at higher oxygen concentrations than do foraminifera; most metazoans begin to respond negatively at ≤1.42 ml/l whereas foraminifera do not respond until oxygen levels are ≤0.5 ml/l [55]–[57]. In addition, species that reproduce only once in a lifetime tend to be better at habitat tracking than those that reproduce in multiple years when environmental changes occur faster than evolutionary responses [58], thus molluscs may be more negatively affected than foraminifera if conditions vary seasonally.

### Body size changes at the population level parallel community-level change

**Figure 3:**
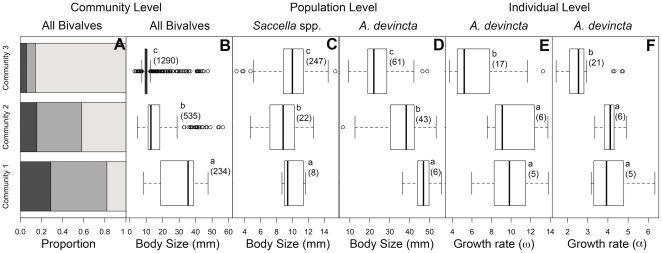
Bivalve body size and growth rate changes over time. (A) Proportion of bivalves that are suspension feeders (dark gray), surface deposit-feeders (medium gray), and subsurface deposit-feeders (light gray), (B) community-level body size, (C) *Saccella* spp. body size, (D) *Anadara devincta* body size, and (E) average growth rate (ω) of *A. devincta* based on external growth breaks and (F) average growth rate (α) of *A. devincta* based on external growth breaks in each of the three molluscan community states. Lower case letters to the upper right of each box plot denotes significant differences (Mann-Whitney, p<0.05). Homogenous groups within each panel are indicated by the same letter. Numbers in parenthesis are the number of individuals represented in each box plot.

Body size at the community-level (bivalves only) declines significantly between subsequent molluscan community states (Mann-Whitney, p<0.001; Figure 3). This corresponds with an increase in the proportional abundance of subsurface deposit-feeding bivalves while both suspension feeders and surface deposit feeders decline (Figure 3). In Community 3, subsurface deposit feeders comprise ∼83% of the community and a single taxon, *Saccella* spp., alone comprises ∼76% of individuals. The decrease in community evenness, increase in dominance of a single taxon, decrease in species richness, and decrease in average body size between molluscan Community 2 and 3 are consistent with an increasingly stressed community [59]–[60]. In environments of high stress, including those with high organic loading or eutrophication, small-bodied opportunistic taxa often bloom and species with r reproductive strategies dominate [53]–[54], [59], [61]–[62]. In the Newport Member, *Saccella* spp. appears to be an “explosive opportunist” as recognized by its aggregation in pods and clusters in thin horizons and its overwhelming dominance in the youngest samples [61]. Much of the body-size change at the community-level can thus be explained by changes in the proportional abundances of species.

In general, the body size at the population level is also predicted to decrease with increased temperature (temperature size rule) or increased environmental stress [59], [63]–[66]. However, species with different ecological requirements are not all stressed by the same factors and can respond individualistically to environmental change sensu [67]. Under conditions of high productivity and organic carbon flux as seen in the Newport Member, subsurface deposit-feeders would find greater food resources and thus may be able to maintain or even increase their body size despite an increase in temperature or accompanying decreases in oxygenation. For example, the deposit-feeding bivalve *Saccella* spp. is significantly larger in Community 3 (n = 247) than in Community 2 (n = 22) (Mann-Whitney, p<0.05; Figure 3). The average body size of subsamples of 22 individuals from Community 3 are significantly greater than Community 2 at the 0.05 level in 87% of cases, indicating that the difference in body size is not due to sample size differences. The shift to larger median body size in *Saccella* spp. from Community 2 to 3 could, however, be due to an increase in the variance of body sizes because minimum sizes decrease while maximum sizes increase (Figure 3). In either case, the body size changes in *Saccella* spp. are not consistent with the hypothesis that body size declines with increasing temperature or stress.

In contrast, body size in the suspension-feeding bivalve *Anadara devincta* is significantly smaller in Community 3 where suspension feeders are rare than in Communities 1 and 2 (Figure 3). Sample sizes are low in Community 1 (6 individuals compared to 43 and 49 in Communities 2 and 3 respectively), but when individuals from Communities 1 and 2 are pooled *A. devincta* from Community 3 remains significantly smaller (Mann-Whitney test, p<0.01). Both maximum and minimum body sizes of *A. devincta* decrease from Community 1 to Community 3, indicating a directional decrease in intraspecific body size (Figure 3). Changes in the size frequency distribution of fossil species can occur if taphonomic loss is size selective; most often it is the smaller individuals that are lost [68]–[69]. However, small-bodied *A. devincta* in Community 1 were probably not lost due to poor preservation or to wave transport because smaller-bodied, thinner-shelled *Saccella* spp. are found in Community 1. Instead, the size shift likely reflects a true change in size-at-death.

The persistence of *A. devincta* in younger strata despite the sharp decline of other suspension feeding taxa is similar to the occurrence of *A. montereyana* from the Miocene Monterey Formation, which is found on bedding planes preserving the dysoxic/oxic boundary [70]. Like the Monterey congener, *A. devincta* may also be adapted to tolerate low oxygen conditions or be able to take advantage of seasonally high oxygen conditions. Living *Anadara* spp., such as *A. ovalis*, have two hemoglobin components, which are thought to be an adaptation to environments with varying oxygen concentrations [71]. Nonetheless, despite this potential adaptive advantage, *A. devincta* body size still declines as productivity and deoxygenation increase through the Newport section, suggesting that individuals are either dying younger or growing slower under stressed conditions. Low-oxygen conditions can reduce growth and feeding in many marine animals and this lowered functional ability is often more pronounced in larger individuals [72]–[73].

In sum, body-size reduction at the community level is driven by both an increase in the abundance of smaller-bodied species and a reduction in size of larger-bodied species. Body size changes also parallel changes in proportional abundance: subsurface deposit-feeders increase in abundance and body size, while suspension feeders decrease in abundance and body size.

### Decreased body size in *Anadara* is driven by decreased growth rates

Measuring changes in growth rates provides a direct test for the influence of individual physiological processes on community and population-level change. Median growth rate is significantly lower in *A. devincta* from Community 3 regardless of the age marker or growth rate metric used (Mann-Whitney, p<0.05; Figure 3; Table S4). Internal and external growth breaks often correspond with δ^18^O maxima (winter low temperatures) suggesting growth breaks are annual, consistent with the extant *A. senilis* in which growth breaks occur annually during the winter [74]. One exceptional fossil specimen of *A. devincta* had an “extra” break between the third and fourth δ^18^O maxima and other specimens had more δ^18^O maxima than growth breaks. Ages estimated from growth break age-markers sometimes differ from ages estimated from geochemical age-markers (e.g., [75]), but in the present study these minor disagreements do not affect the consistent pattern of declining growth rates over the time series.

Both the maximum and minimum growth rates observed in *A. devincta* specimens decrease from Community 1 to Community 3 indicating that the decrease in median growth rate across communities is driven by individuals growing slower (Figure 3). Some individuals from the oldest Community 3 beds have growth rates comparable to Communities 1 and 2, suggesting that the environmental changes that drive the shift in community composition from Community 2 to Community 3 did not negatively affect *A. devincta* as immediately as they did the suspension and surface deposit-feeders that went locally extinct. This lag in responses by *A. devincta* supports the hypothesis that it was fundamentally better adapted to low-oxygen and high-productivity conditions than were other suspension feeders.

Paleoecological studies of mass extinctions find reduced body sizes in surviving taxa and have termed this phenomenon the “Lilliput Effect” [76]–[77]. For example, the body size reduction following the late Permian mass extinction appears to have been driven by reduced overall growth rates and more frequent interruptions to growth, which have been attributed to environmental factors such as dysoxic episodes, extreme temperature events, or low productivity [78]. In modern systems, reduced growth rates or reduced “size-at-age” in ectotherms are associated with exposure to increased temperature and concurrent reductions in oxygen availability [66], [79]. Often it is this combined thermal and oxygen stress that demands physiological adaptation, making predictions of faunal responses based on temperature alone difficult [80]. The Newport Member provides an example of reduced growth rates in a bivalve over a period of global warming that may also have been driven by low-oxygen stress as its proximal cause. Other environmental variables that could retard growth rate include low food quality and predator density but these are not testable in the Newport Member.

Parallel changes between physiological rates and community properties are generally taken as signifying that changes at the community-level emerge from processes operating at the individual-level [81]. In the case of the Newport Member, declines in the proportional abundance of suspension feeders and in community-level body size are paralleled by decreased body size and decreased growth rates in the suspension feeder *A. devincta*, a numerically important component of the community. Thus, the physiological tolerance of individuals provides a mechanistic link to community-level change in the Newport Member, and changes in community composition and structure did not arise simply from changes in propagule supply.

The decline in *A. devincta*'s physiological health also implies that this species did not respond evolutionarily to the new environmental conditions despite having ∼1 Ma to adapt. This suggests that the environmental stress was imposed on only part of its geographic range, such that gene flow among stressed and unstressed populations prevented local adaptation; *A. devincta* is known to range from Alaska to Southern California during the Early Miocene [24], [82]. Alternatively, the decrease in body size could be a locally adaptive paedomorphic response to environmental stress, which would be accompanied by an earlier onset of reproductive maturity marked by an earlier ontogentic decline in growth rates [83], [84]. In contrast, the age at which growth rates decline most sharply in *A. devincta* is consistent across the three communities (age-marker 4), indicating that the paedomorphic hypothesis can be rejected. Additional sclerochronological examination of shells for reproductive growth checks might reveal the onset of reproduction. However, in the six shells for which both geochemical age-markers and growth line age-markers are available, reproductive breaks are rarely preserved and there is no evidence for an ontogenically younger onset of reproduction to suggest that paedomorphosis played a role.

### Implications for predicting future ecosystem change

Abrupt changes across an environmental threshold are common in modern ecology and receive much attention because they affect our ability to predict how and when biota will respond to environmental drivers. Abrupt changes can also indicate shifts to new, persistent, ecological regimes and are often caused by a combination of climate changes and anthropogenic impacts [13], [85]. Such “threshold shifts” with eutrophication are recognized in modern, anthropogenically-influenced ecological systems and in large marine ecosystems with overfishing [14], [85]–[86]. Threshold shifts have also been observed in pelagic marine systems associated with anthropogenic warming and ice melt in the North Atlantic, which may or may not be influenced by fishing in the region [87]–[88]. Threshold shifts with respect to environmental variables in a pre-anthropogenic ecological record over intervals <0.1–0.3 Ma suggest that rapid changes due human influences are not necessary to push ecosystems over a threshold: climate changes occurring at natural rates and within the range of natural variability are sufficient.

The Newport Member also demonstrates that the proximal driver of ecological changes during a global warming event can be related to changes in productivity and oxygenation in some settings, as in areas of upwelling, instead of temperature itself. Identifying the particular environmental variables that drive ecological change is integral to anticipating which taxa or functional groups will most likely be affected. In addition, if changes in productivity typically drive ecological responses to global warming in coastal marine environments, then we may be late in detecting changes in many modern systems that are already eutrophied, either naturally or from human activities in the watershed. The life history characteristics of taxa also influence how they respond to environmental changes and, thus, the predictions derived from studying one group cannot necessarily be extrapolated to others. This study highlights the power of examining multiple environmental variables and multiple taxonomic groups on multiple biological levels in order to develop better predictive frameworks for biotic response to climate change.

Although fossil records are usually limited to skeletal hard parts preserved in time-averaged assemblages, information on physiological rates can be extracted from growth rate analyses and, in that way, mechanistic links can be drawn between the environmental tolerances of individuals and changes at the community level. These links strengthen confidence in identifying the particular environmental factors driving change and also provide a physiological basis for predicting the most vulnerable taxa. Bivalve growth rates in living populations are already used to detect physiological stress during anthropogenically driven environmental deterioration (e.g., [89]–[90]). The present study shows that bivalve growth rates can also be instrumental in detecting individual responses to fully natural environmental changes on a range of time scales. Growth rate analysis is thus a potential tool for detecting the early stages ecological change in modern systems, before change is manifested at the community level.

Fossil records with good temporal resolution that span past climate change events, like that provided by the Newport Member, can yield detailed information on (a) the tempo of paleoecological changes, (b) the environmental drivers of those changes, and (c) and the effects on individual physiology. This high level of detail is invaluable to forecasts of modern ecosystem change as the climate system moves outside the realm observed by modern ecology, and perhaps into no-analog regimes known only from the deeper geological record. Systematic exploration of fossil records that span histories of environmental change and ecosystem response will illuminate the conditions that induce regime shifts and the rates at which such abrupt shifts between community states can occur. In addition, targeting environments, like coastal settings, that are especially vulnerable to modern global warming will heighten the value that paleoecological analysis brings to present ecological challenges.

## Materials and Methods

Sediment samples were collected from fresh outcrop surfaces for benthic foraminifera sampling at a resolution of ∼1 sample/m of stratigraphic section. Approximately 100 g of sediment from each sample were disaggregated in ultrapure water before wet-sieving through a 150 mm nylon sieve. The total sample was picked if it contained <300 individuals >150 mm. Richer samples were split using a microsplitter to obtain ∼300 individuals each. The minimum sample size included in analyses was 100 individuals. Bulk samples for molluscan sampling were extracted from nineteen stratigraphic beds exposed in outcrop or from fallen blocks whose original stratigraphic position was clear for a resolution of ∼1 sample/10 m. The minimum molluscan sample size included in analyses was 85 individuals

Molluscan samples are drawn from the total volume of the bed, regardless of bed thickness. The smallest molluscan individual identified was 4 mm. For thicker units, multiple samples are taken from different vertical levels within the bed for foraminiferal faunal analysis so that within bed changes are recorded. All necessary permits were obtained for the described field studies from the Oregon Parks and Recreation Department.

For each faunal sample, species richness (sample-size standardized to the minimum number of specimens), evenness (PIE following Hurlbert, 1971) [91], and the proportional abundances of ecological groups are calculated. Detrended correspondence analysis (DCA) is performed on the proportional abundances of species using the decorana function in the vegan package in the R programming language [92]–[93]. Non-metric multidimensional scaling (NMDS) is performed on the Bray-Curtis distances among samples based on the proportional abundances of species using the isoMDS function in the MASS package in the R programming language [92]. Correlations between ordination score and environmental variables are preformed on the axis that summarizes the greatest proportion of variance. Axis 1 summarizes 63% of the variance in DCA and 73% in NMDS (stress = 5.86; 3 dimensions) for the molluscan analysis and 65% and 56% (stress = 9.04; 3 dimensions) respectively for the foraminiferal analyses. The proportion of variance in the data set summarized by each ordination axis is computed via a Mantel Test on the Euclidean distances between points and the Bray-Curtis distances between faunal assemblages in the unreduced space (following [94]).

For the bivalves, species are categorized as suspension-feeders, surface deposit-feeders, or sub-surface deposit-feeders based on the feeding preferences of living congeners. For the foraminifera, the proportional abundance of taxa tolerant of low oxygen and high organic sediments (buliminids, *Fursenkoina* spp. and *Nonionella* spp.) is calculated. Molluscan body size is also measured at the community level and is calculated as the square root of the product of valve length and height (geometric mean size) e.g. [95]. The tempo of changes in the above ecological metrics are tested using maximum likelihood model selection fitting to four models of temporal change: no change, change without directional bias, directional change, and stepped change. Procedures follow Hunt [96]–[97] and use the paleoTS package in the R programming environment [92].

At the population level, body size is examined using 110 *Anadara devincta* and 277 *Saccella* spp. shells from bulk samples and from opportunistic outcrop collections. Growth rates were only examined in *A. devincta*: this species has a thick shell with well-preserved growth banding unlike *Saccella* whose valves <1 mm thick. Size-at-age was estimated using (1) external growth lines, (2) internal growth lines, and (3) δ^18^O maxima along the growth axis as annual markers of winter temperatures. Collectively, these are referred to here as “age markers.”

Prominent external growth lines were measured on 32 well-preserved individuals of *A. devincta* with dial calipers to the nearest 0.1 mm. All measurements were made from the umbo along the maximum growth axis of the shell. Bivalve shells were then coated in epoxy and cross-sectioned along the maximum growth axis using a low-speed saw. Shell cross-sections were ground on glass plates (320 and 600 grit powder), polished with 1200 grit pat gel, and sampled for stable isotope analysis with a 300 mm drill bit. Cross-sections with visible internal growth lines (14) were digitally scanned and internal growth lines were measured from a fixed point on the umbo to the intersection of the growth line with shell edge using the software Image J (NIH) to the nearest 0.01 mm. From six shells, 12–34 microsamples were taken along the maximum growth axis for a resolution of ∼1 sample every 0.75 mm for stable isotope analysis. Shell powders were analyzed at the University of Michigan Stable Isotope Laboratory on a Finnigan MAT 251 mass spectrometer for δ^18^O and δ^13^C. Analytical precision is 0.06‰ for δ^18^O. Prior to stable-isotope sampling, preservation of original aragonite was confirmed using x-ray diffraction for mineral composition and scanning-electron microscopy for mineral structure. The δ^18^O maxima (cool temperatures) were chosen as age markers instead of δ^18^O minima because the winter values form sharp cusps in plots of the δ^18^O values in the direction of growth whereas summer values form broader plateaus and thus yield less precise size-at-age estimates.

Growth rates for individual shells were calculated from size-at-age for each age-marker method in two ways: (a) using the slope of the linear regression (α) of the first 5 age markers and (b) fitting the von Bertalanffy growth function to all age markers. The distance from the umbo for each age marker were fitted to a growth curve using the von Bertalanffy growth function and growth rate (ω) is defined from the model parameters following Jones et al. [98]. Omega (ω) is representative of the growth rate near t_0_. Differences in α and ω between communities are tested using a Kruskal-Wallis test and post-hoc Mann-Whitney tests.

The position and number of internal growth increments and δ^18^O maxima are also compared. If internal growth increments occur more frequently than δ^18^O maxima, these additional increments may be reproductive growth checks and thus indicate reproductive maturity.

Strontium isotope ages were obtained from molluscan carbonate from 12 locations and have a precision of ±0.23 Ma (Table S1) [30]. Constant sedimentation rates are assumed between Sr-isotope ages to assign intervening faunal samples to an age. Sr-isotope ages from each stratigraphic section along the coast combined with lithologic marker beds allow faulted blocks to be placed in correct temporal order and for repeated sections to be identified. All stratigraphic sections have portions of overlap, which allow them to be joined in a composite section [30].

Four environmental proxies from the Newport Member were compared to faunal patterns to identify potential environmental drivers of faunal changes including temperature (δ^18^O), water column productivity (δ^13^C), organic carbon flux (Δδ^13^C), and percent mud (see Table 5 for summary of all paleoenvironmental data types considered in this study). The δ^18^O and δ^13^C measurements were made on *Buccella mansfieldi*, a semi-infaunal benthic foraminifer. Temporal trends in δ^18^O are consistent among the three foraminiferal taxa and among fault repeated stratigraphic sections, but there are clear offsets in δ^13^C among the taxa consistent with life habit differences indicating that original calcite is preserved [30]. The Δδ^13^C was calculated as the difference between the δ^13^C values of *B. mansfieldi* and *Pseudononion costiferum*, an infaunal foraminifer. Mud content was measured as the weight percent of sediment grains <63 mm [30]. Trace element analyses were also performed on a limited number of samples to reconstruct paleotemperature (Mg/Ca) and productivity (Cd/Ca), but samples were judged to be contaminated by siliciclastic material.

**Table 5:** Environmental variables measured in Newport Member of the Astoria Formation.

Environmental Variable	Source	Environmental Proxy
Temperature	Foraminifera	δ^18^O, Mg/Ca*
Productivity	Foraminifera	δ^13^C, Cd/Ca*
Organic Matter Flux 1	Foraminifera	Δδ^13^C
Organic Matter Flux 2	Foraminifera, Molluscs	habitat of modern congenerics
Sediment Grain Size	Sediments	% mud
Seasonality*	Bivalve	δ^18^O along maximum growth axis
Freshwater flux	Foraminifera, Sediments	δ^18^O, max grain size
Water depth	Foraminifera, Molluscs, Sedimentology	habitat of modern congenerics, grain size, sedimentary structures
Oxygenation	Foraminifera, Molluscs, Sedimentology	sedimentary laminations, habitat of modern congenerics

Source indicates the materials measured for each variable.

* = Variables or proxies not measured in the present study, which have the potential to yield additional information after methodological improvements.

## Supporting Information

Figure S1 **Boxplots of horizontal distributional mean calculations of modern equivalent latitude for molluscan communities 1–3 showing similar estimated latitudes for each community.** For each community pool, the horizontal distributional mean characteristic curve (HDM) of the modern latitudinal ranges of constituent genera is calculated following [99]. The median of the curve is taken as the modern equivalent latitude of the sample. Species abundances from the pooled communities are bootstrap resampled to 300 individuals for each calculation and resampling is repeated 1000 times to test for sampling effects in the result. Heavy horizontal bar is the median latitude from the resampling procedure, the box encompasses the interquartile range, whiskers extend to the most extreme data point within 1.5 times the interquartile range, and open circles denote data outside 1.5 times the interquartile range.
(TIF)

Table S1 **Sampling coverage with meters above base of composite stratigraphic section and ages for each sample.** F = foraminiferal faunal sample; M = molluscan faunal sample; S = sediment grains size (% mud); SI = stable isotope (δ^18^O and δ^13^C); Sr = Strontium isotope, G = bivalve growth. The first 2–3 letters in the sample code indicate the stratigraphic section and the number indicates the bed from which the sample was obtained. Letters following the bed number differentiated samples that were taken at different levels within the same bed. YH = Yaquina Head, NYF = North Yaquina, DM = Moolach Beach, SE = Section E, SP = Schooner, RR = Reef Rocks. These names correspond to section labels in the stratigraphic log published as Figure 1 in [30]. The Schooner-Yaquina column contains, in order from oldest to youngest, SP, RR, NYF, and YH.
(CSV)

Table S2 **AIC values, AICc values, and Akaike weights based on AICc values for five models of changes in species diversity, PIE, the proportion of ecologically important taxa: URW (unbiased random walk), GRW (general random walk), stasis, 1-Shift, and 2-Shifts.** “n” is the number of samples in the time series. “Shift start” indicates the age of the youngest sample prior to shift.
(TXT)

Table S3 **Partial correlations based on Spearman rank order between foraminiferal ecological and environmental variables where the resolution of the foraminiferal data set has been reduced to match the resolution of the molluscan data set.** None of the partial correlations are significant.
(DOC)

Table S4 **Median growth rates for** ***Anadara devincta*** **in each community calculated as the slope of a linear regression and as the parameter ω derived from the von Bertalanffy growth equation for external growth increments, internal growth increments, and δ^18^O maxima.** Krustal-Wallis (K-W) and Mann-Whittney (M-W) tests report which communities have significantly different growth rates. Homogeneous groups (p>0.05) in pairwise tests are indicated by the same letter (A, B). Communities 1 and 2 are also combined due to small sample sizes in these two communities. IQR = interquartile range, N = number of individuals.
(DOC)
