# Supplementary figures and images for: Individual to Community-Level Faunal Responses to Environmental Change from a Marine Fossil Record of Early Miocene Global Warming
Source: PLoS One. 2012 Apr 27;7(4):e36290. doi: 10.1371/journal.pone.0036290 (PMC3338691; doi:10.1371/journal.pone.0036290)

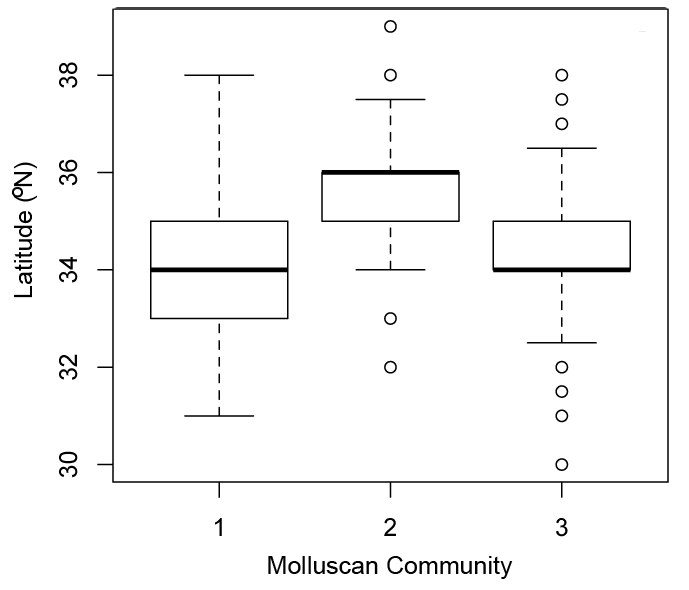

Supplement: Figure S1 — Boxplots of horizontal distributional mean calculations of modern equivalent latitude for molluscan communities 1–3 showing similar estimated latitudes for each community. For each community pool, the horizontal distributional mean characteristic curve (HDM) of the modern latitudinal ranges of constituent genera is calculated following [99]. The median of the curve is taken as the modern equivalent latitude of the sample. Species abundances from the pooled communities are bootstrap resampled to 300 individuals for each calculation and resampling is repeated 1000 times to test for sampling effects in the result. Heavy horizontal bar is the median latitude from the resampling procedure, the box encompasses the interquartile range, whiskers extend to the most extreme data point within 1.5 times the interquartile range, and open circles denote data outside 1.5 times the interquartile range. (TIF) [file pone.0036290.s001.tif]
