# Supplementary material for: Individual to Community-Level Faunal Responses to Environmental Change from a Marine Fossil Record of Early Miocene Global Warming
Source: PLoS One. 2012 Apr 27;7(4):e36290. doi: 10.1371/journal.pone.0036290 (PMC3338691; doi:10.1371/journal.pone.0036290)
Supplement: Table S3 — Partial correlations based on Spearman rank order between foraminiferal ecological and environmental variables where the resolution of the foraminiferal data set has been reduced to match the resolution of the molluscan data set. None of the partial correlations are significant. (DOC) [file pone.0036290.s004.doc]

|  | Richness | Evenness | NMDS 1 | DCA 1 |
| --- | --- | --- | --- | --- |
| δ^18^O | -0.522 | -0.423 | 0.433 | -0.568 |
| δ^13^C | 0.099 | 0.151 | -0.435 | 0.588 |
| Δδ^13^C | -0.053 | -0.175 | -0.245 | 0.316 |
| % mud | 0.447 | 0.201 | -0.207 | 0.359 |
