# Supplementary material for: Individual to Community-Level Faunal Responses to Environmental Change from a Marine Fossil Record of Early Miocene Global Warming
Source: PLoS One. 2012 Apr 27;7(4):e36290. doi: 10.1371/journal.pone.0036290 (PMC3338691; doi:10.1371/journal.pone.0036290)
Supplement: Table S4 — Median growth rates for Anadara devincta in each community calculated as the slope of a linear regression and as the parameter ω derived from the von Bertalanffy growth equation for external growth increments, internal growth increments, and δ18O maxima. Krustal-Wallis (K-W) and Mann-Whittney (M-W) tests report which communities have significantly different growth rates. Homogeneous groups (p>0.05) in pairwise tests are indicated by the same letter (A, B). Communities 1 and 2 are also combined due to small sample sizes in these two communities. IQR = interquartile range, N = number of individuals. (DOC) [file pone.0036290.s005.doc]

| Method |  | Linear Regression | | | | von Bertalanffy Best Fit | | | |
| --- | --- | --- | --- | --- | --- | --- | --- | --- | --- |
| External Growth Increments | Community | Median Slope | IQR | n | K-W (p=0.0005) | Median Omega | IQR | n | K-W (p=0.0152) |
|  | 1 | 3.93 | 3.30-4.78 | 5 | 3 | 9.88 | 8.27-11.50 | 5 | 3 |
|  | 2 | 4.12 | 3.88-4.27 | 6 | 3 | 9.1 | 8.45-11.67 | 6 | 3 |
|  | 1+2 | 4.06 | 3.59-4.54 | 11 | 3 | 9.52 | 8.32-11.94 | 11 | 3 |
|  | 3 | 2.54 | 2.10-2.81 | 21 | 1,2 | 5.2 | 4.49-7.82 | 17 | 1,2 |
| Internal Growth Increments | Community | Median Slope | IQR | n | M-W (p=0.0024) | Median Omega | IQR | n | M-W (p=0.0051) |
|  | 1+2 | 2.87 | 2.46-3.27 | 6 | 3 | 15.66 | 10.85-22.69 | 6 | 3 |
|  | 3 | 1.57 | 1.40-1.75 | 8 | 1+2 | 4.84 | 3.77-6.19 | 6 | 1+2 |
| δ^18^O maxima (winters) | Community | Median Slope | IQR | n |  | Median Omega | IQR | n |  |
|  | 1+2 | 3.30 | 2.98-3.54 | 4 | --- | 8.37 | 7.14-9.15 | 3 | --- |
|  | 3 | 1.60 | 1.60-1.67 | 2 | --- | 3.33 | 2.80-3.86 | 2 | --- |
